# The Role of XBP1 in bone metabolism

**DOI:** 10.3389/fendo.2023.1217579

**Published:** 2023-09-19

**Authors:** Wenhao Lv, Youli Zheng, Junjun Jiao, Yu Fu, Tingrui Xu, Li Zhang, Zheng Zhang, Ning Ma

**Affiliations:** ^1^ Hospital of Stomatology, Jilin University, Changchun, China; ^2^ The School and Hospital of Stomatology, Tianjin Medical University, Tianjin, China; ^3^ Tianjin Stomatological Hospital, School of Medicine, Nankai University, Tianjin, China; ^4^ Tianjin Key Laboratory of Oral and Maxillofacial Function Reconstruction, Tianjin, China

**Keywords:** XBP1, osteoclast, osteoblast, immune microenvironment, bone metabolism

## Abstract

Bone is a dynamic organ that, once formed, undergoes a constant remodeling process that includes bone resorption and synthesis. Osteoclasts and osteoblasts are primarily responsible for controlling this process. X-box binding protein 1 (XBP1), a transcription factor, affects the metabolism of bones in various ways. In recent years, numerous studies have revealed that XBP1 plays a vital role in bone metabolism, including osteoclast and osteoblast development, as well as in regulating immune cell differentiation that affects the immune microenvironment of bone remodeling. In this review, we highlight the regulatory mechanisms of XBP1 on osteoclasts and osteoblasts, how XBP1 affects the immune microenvironment of bone remodeling by influencing the differentiation of immune cells, and predict the possible future research directions of XBP1 to provide new insights for the treatment of bone-related metabolic diseases.

## 1 Introduction

Bone is a rigid and dynamic organ that experiences a phenomenon referred to as remodeling (1, 2). In a typical state, there exists an equilibrium between the creation and breakdown of bone, overseen by osteoblasts and osteoclasts, respectively (3). Nevertheless, when these mechanisms become imbalanced, they can result in significant impairments to the structure and functionality of bone, consequently giving rise to various bone metabolic ailments, including osteoporosis, rheumatoid arthritis, and multiple myeloma (MM) (4–7).

Throughout the process of skeletal development, osteoblasts generate substantial quantities of extracellular matrix proteins. Endoplasmic reticulum stress (ER stress) is elicited by both endogenous and exogenous stimuli, leading to the activation of the unfolded protein response (UPR) (8, 9). In mammalian cells, three transmembrane proteins located in the endoplasmic reticulum serve as sensors for ER stress: protein kinase R-like endoplasmic reticulum kinase (PERK), activating transcription factor 6 (ATF6), and inositol-requiring enzyme 1α (IRE1α) (10). These proteins mediate the three branches of UPR signaling, namely PERK-eIF2α, ATF6, and IRE1α (11). Among these branches, the IRE1α/XBP1 pathway represents one of the most highly conserved signaling pathways throughout evolution (12). When IRE1α is activated, it facilitates the cleavage of unspliced XBP1 mRNA into spliced XBP1, which subsequently participates in the regulation of endoplasmic reticulum homeostasis, cell survival, angiogenesis, and other biological processes (13–15).

As a transcription factor, x-box binding protein 1 (XBP1) assumes a crucial function in the immune microenvironment of bone remodeling by promoting osteoclast formation (8, 16–18), osteoblast differentiation (19), and immune cell differentiation through diverse cytokines or signaling pathways (20).

XBP1, a key transcription factor, is crucial in the response of unfolded proteins under ER stress conditions and is associated with the pathogenesis of bone metabolic diseases (16, 21). Therefore, understanding the regulation of XBP1 in bone metabolism is crucial and may lead to new advances in treating various bone metabolic diseases. Current research on XBP1 has focused on the regulatory role in various tumor diseases, and little is known about its role in bone metabolic diseases. Herein, we summarize the critical roles of XBP1 in the differentiation of osteoclasts, osteoblasts, and immune cells, as well as in influencing the immune microenvironment of bone remodeling. In addition, the review also predicts possible future research directions of XBP1, providing new ideas for the treatment of various diseases.

## 2 XBP1

It is a distinctive transcription factor of base-region leucine zipper (bZIP) that governs the regulation of human MHC class II genes (22, 23). Subsequent to this discovery, numerous investigations conducted approximately ten years later have demonstrated that XBP1 is a downstream component of IRE1α, a crucial transcription factor for the UPR in both invertebrates and vertebrates, and is indispensable for cellular survival in reaction to stressful stimuli (14, 24).

XBP1 can be produced by plasma cells (PCs), MM cells, and bone marrow stromal cells (BMSCs) (25, 26). XBP1 is highly expressed in PCs and alleviates ER Stress through UPR. XBP1 can also be induced in BMSCs in the MM microenvironment. Guoshuang Xu et al. found that XBP1 mRNA levels are significantly increased in BMSCs from MM patients compared to healthy donor. Meanwhile, they found that BMSCs with elevated levels of XBP1 protein induced more osteoclast formation (16).

XBP1 plays a role as a transcription factor not only in osteoclast differentiation but also in osteoblast differentiation. Initially, only literature using Northern-blot analysis demonstrated that XBP1 expresses during osteoblast differentiation, which PTH influences (27). A couple of years later, Takahide Tohmonda performed *in vitro* osteoblast differentiation assays using mouse embryonic fibroblasts (MEFs) and recombinant bone morphogenic protein 2 (BMP2) to detect the expression of early and late markers of osteoblast differentiation. These experimental results demonstrate that the IRE1α/XBP1 pathway is essential for BMP2-induced osteoblast differentiation (19).

In macrophages, the promotion of inflammatory cytokine production and M1-type macrophage polarization by XBP1 may play a role in osteoclast differentiation (28). Conversely, the activation of IRE1α/XBP1 signaling by estrogen can inhibit M1-type macrophage polarization (29). In CD4+ T cells, XBP1 can contribute to Th-cell polarization, leading to the secretion of various cytokines that impact osteoblast or osteoclast differentiation processes. Additionally, XBP1 in CD8+ T cells can regulate the expression of CD8+ T cell inhibitory receptors induced by cholesterol and induce T cell exhaustion. Furthermore, the activation of XBP1 by SIRT7 has been observed to selectively regulate the expression and secretion of cytokines, thereby exerting an influence on the process of bone remodeling. Within B cells, the inhibition of osteoclastogenesis can be achieved through the secretion of osteoprotegerin (OPG), which blocks the RANKL/RANK pathway. However, in MM PCs, the potential to promote osteoclastogenesis exists (30). Although XBP1 is abundantly expressed in PCs, the precise mechanism through which it impacts bone metabolism in this cell type remains unclear. It may affect the immune microenvironment of bone remodeling by influencing PCs differentiation and immunoglobulin (Ig) secretion.

**Figure 1:**
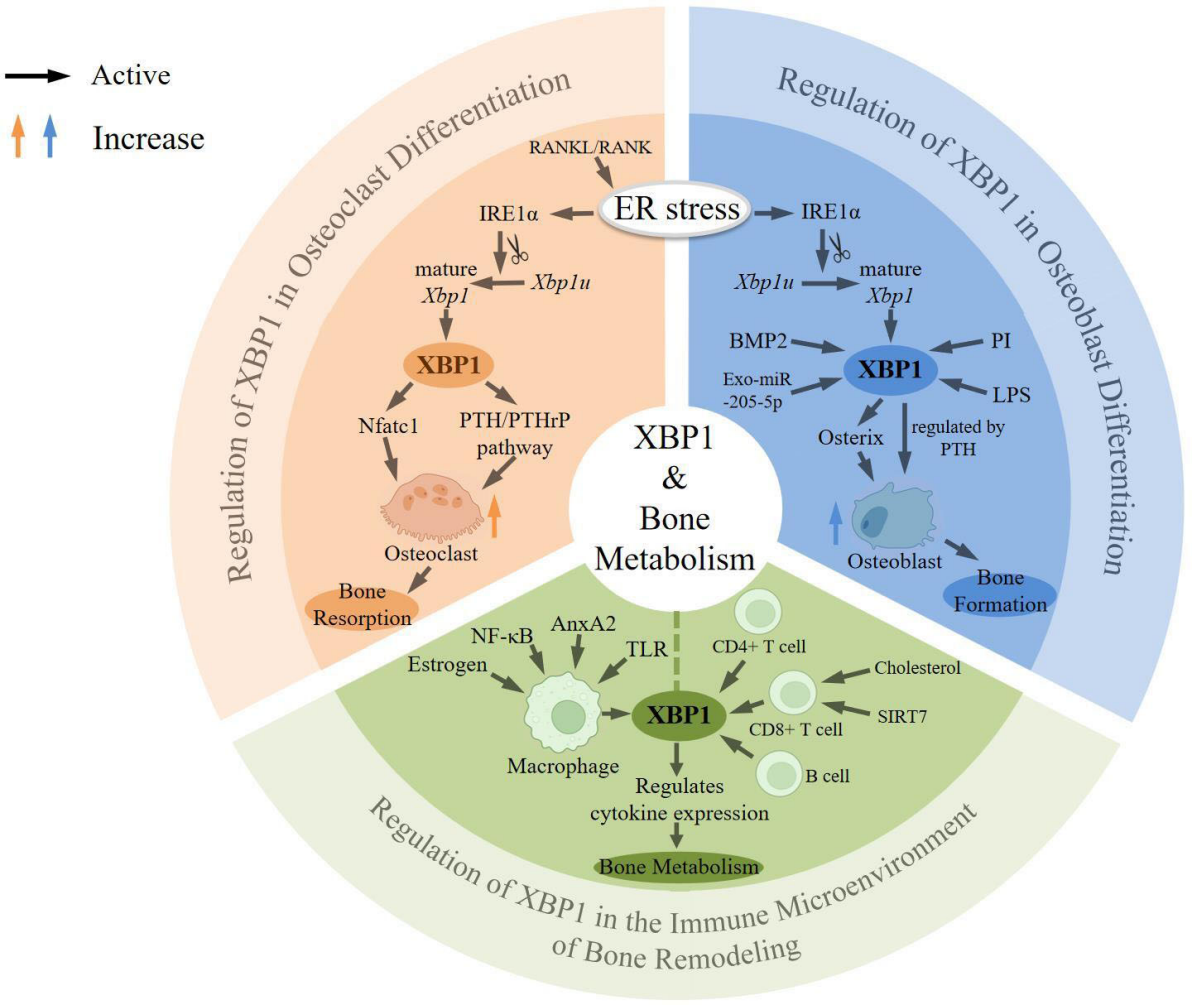
Overview of the role of XBP1 in bone metabolism. During the process of osteoclast differentiation, the activation of endoplasmic reticulum stress by RANKL/RANK leads to the activation of IRE1α, resulting in slicing Xbp1u into mature Xbp1. X-box binding protein 1 (XBP1) can induce the formation of osteoclasts by promoting the transcription of Nfatc1 or by positively regulating the expression of the PTH/PTHrP pathway, which subsequently leads to bone resorption. Conversely, during osteoblast differentiation, XBP1 can be activated by various factors including proteasome inhibition (PI), bone morphogenic protein 2 (BMP2), lipopolysaccharide (LPS), and specific miRNAs such as Exo-miR-205-5p. XBP1 regulates the expression of Osterix, thereby facilitating osteoblast differentiation and bone formation, regulated by PTH. In macrophages, XBP1 can be activated by toll-like receptors (TLR), Annexin A2 (AnxA2), NF-κB, and estrogen. In T cells and B cells, XBP1 is highly expressed and thus may regulate bone metabolic processes. Yellow arrows represent increased osteoclastogenesis and blue arrows represent increased osteoblastogenesis.

Next, the review will discuss the role of XBP1 in regulating osteoclast differentiation, osteoblast differentiation, and affecting the immune microenvironment of bone metabolism, respectively (**Figure 1**).

## 3 Regulation of XBP1 in osteoclast differentiation

### 3.1 XBP1 is involved in osteoclast formation via the IRE1α/XBP1 pathway in the UPR branch

The researchers conducted *in vitro* experiments to investigate the formation of osteoclasts using wild-type (WT) bone marrow macrophages (BMMs), recombinant soluble RANKL (sRANKL), and murine CSF1. The results demonstrated that the differentiation of osteoclasts under physiological conditions led to transient induction of ER stress (17). To further explore this phenomenon, the authors employed siRNA-mediated gene silencing to inhibit the IRE1α/XBP1 pathway in WT BMMs and conducted *in vitro* assays to assess osteoclast formation in these cells. The experimental results revealed that the IRE1α/XBP1 pathway plays a critical role in the regulation of osteoclast differentiation and that the termination of this pathway significantly inhibited osteoclast formation *in vitro* (17, 31).

**Figure 2:**
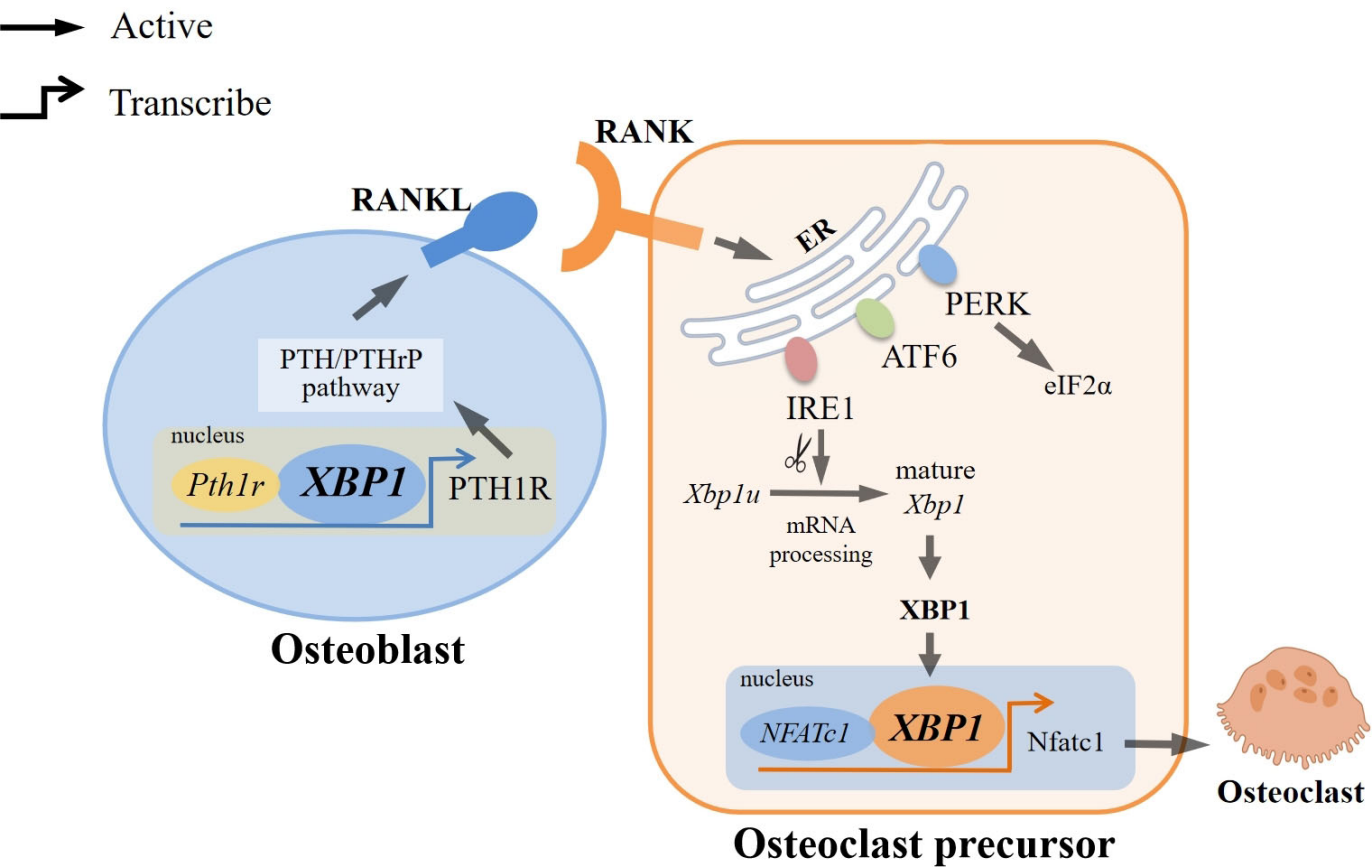
Regulation of XBP1 in Osteoclast Differentiation. RANKL/RANK can trigger endoplasmic reticulum stress (ER stress), which activates three typical branches of the unfolded protein response: PERK-eIF2α, ATF6, and IRE1α. Among them, IRE1α/XBP1 is the most evolutionarily conserved pathway, and IRE1α can splice *Xbp1u* into mature *Xbp1*. XBP1 can directly bind to the binding sites of *NFATc1*, a major regulator associated with osteoclast formation, to induce transcription of Nfatc1, thereby promoting osteoclast formation. In addition, XBP1 can also bind directly to the promoter of *Pth1r* to positively regulate PTH/PTHrP expression and participate in PTH-induced RANKL expression, which interacts with RANK, thereby inducing differentiation of osteoclast precursor into osteoclast.

The researchers discovered that the involvement of IRE1α/XBP1 in osteoclast formation can be attributed to a series of processes. The activation of IRE1α, triggered by ER stress induced by RANKL/RANK, leads to the splicing of XBP1u into mature XBP1. Notably, the *Nfatc1* plays a crucial role in regulating osteoclast formation, and its promoter region contains potential binding sites for XBP1. Consequently, the direct binding of mature XBP1 to these sites stimulates the transcription of Nfatc1, thereby facilitating the promotion of osteoclast formation (**Figure 2**). Moreover, it was observed that ER stress was only transiently induced following RANKL stimulation, indicating that the role of the UPR in maintaining mature osteoclast function is limited (17, 32).

Additionally, a substantial body of evidence has demonstrated that BMMs lacking IRE1α exhibit impaired ability to induce Nfatc1 after RANKL stimulation (17). Furthermore, in a study conducted by Lavinia Raimondi et al., pre-osteoclast cells (pOCs) were treated with MM cell-derived small extracellular vesicles (MM-EVs), revealing an increased expression of XBP1 in pOCs after 3 days of treatment with MM-EVs compared to untreated cells. Subsequently, the researchers proceeded to suppress the IRE1α/XBP1 pathway by employing a specific inhibitor of IRE1α. Remarkably, the application of MM-EVs in conjunction with the IRE1 inhibitor led to a significant decrease in the quantity of osteoclasts within Raw264.7 cells (33). These findings serve to further validate the significance of the IRE1α/XBP1 pathway in the process of osteoclast formation.

### 3.2 XBP1 is involved in osteoclast formation through regulation of the PTH/PTHrP pathway

Furthermore, it has been postulated that parathyroid hormone (PTH) and parathyroid hormone-related protein (PTHrP) exert pivotal regulatory functions in bone development and homeostasis. The transcription of the PTH/PTH-related peptide receptor (PTH1R) is regulated by the IRE1α/XBP1 pathway, as evidenced by the direct interaction of XBP1 with the P2 promoter of *Pth1r*. Consequently, the IRE1α/XBP1 pathway exerts a positive influence on the expression of the PTH/PTHrP pathway, ultimately facilitating the upregulation of PTH-induced RANKL expression in osteoblasts. This, in turn, leads to the activation of osteoclastogenesis through the interaction between RANKL and RANK on osteoclasts (8, 34).

## 4 Regulation of XBP1 in osteoblast differentiation

### 4.1 XBP1 regulates osteoblast differentiation by promoting the expression of Osterix

Osterix (Osx) is a novel zinc finger-containing transcription factor specifically expressed in all growing bones. It is viewed as a transcription factor essential for osteoblast differentiation (35). Without Osx, there is no cortical and trabecular bone formation via intramembranous or intracartilaginous osteogenesis. In the progression of osteoblast differentiation, osteoblast progenitors in intrachondral and interstitial cohesion of membranous bone elements differentiate into preosteoblasts through regulation by Runx2/Cbfa1, which in turn differentiate into mature osteoblasts in one or more steps and express characteristic osteoblast marker genes, a process that requires Osx (36, 37).

Takahide Tohmonda et al. discovered through several *in vitro* experiments that the osteoblast-specific transcription factor Osx is the direct target of XBP1, not Runx2. Researchers cultured multipotent, uncommitted MEFs and committed preosteoblasts *in vitro* and found that XBP1 regulates Osx expression by binding directly to the XBP1 binding site in the Osx promoter (38). Besides, using UPR signaling released by thapsigargin, a potent inducer of ER stress, could promote Osx transcription in BMP2-treated fibroblasts and osteoblast cell line MC3T3-E1, but this process was dependent on IRE1α/XBP1 (19). In addition, it has been shown that mutant reporter constructs analysis and a luciferase assay, based on data from the promoter region of Osx encoding a UPR element (TGACGTGG/A)-like sequence (TGAGCTGG) (39)and XBP1-binding sequence (ACGT) (40), reveals that the IRE1α/XBP1 pathway stimulates Osx expression and thus promotes the differentiation of preosteoblasts into osteoblasts (19).

### 4.2 XBP1 can be activated by proteasome inhibition and involved in bone formation

Over the past decade, the medical community has introduced several new drugs for treating MM, of which proteasome inhibition (PI) is the most important class. PI has been shown to promote bone formation *in vivo* and vitro (41). Three PIs have been approved for the treatment of MM: bortezomib, carfilzomib, and ixazomib (42), which can reduce bone resorption and increase bone formation in MM patients (43, 44). Studies have shown that bortezomib counteracts the osteoclast regulators, RANKL and OPG, leading to osteoclast inhibition and reduced bone destruction. It also stimulates osteoblast differentiation, as evidenced by elevated bone-specific alkaline phosphatase (BSP) and osteocalcin (OCN) (45).

*In vitro* studies have provided evidence that myeloma cells possess the ability to hinder the differentiation of osteoblast precursors and trigger apoptosis in fully developed osteoblasts (46). A particular study demonstrated that the administration of bortezomib resulted in the expression of markers associated with osteogenesis, as observed through the assessment of calcium deposition in bortezomib-treated mouse BMSCs using Alizarin Red S (ARS) staining (47). Recent investigations involving ARS staining and Western blot analysis of IRE1α inhibitor-treated bortezomib-induced MSCs and MC3T3-E1 cells have indicated that the inhibition of XBP1 disrupts the osteogenic differentiation process induced by bortezomib (44). Moreover, the study revealed that the upregulation of XBP1 in MSCs and MC3T3-E1 cells through the utilization of Tet-On-inducible lentiviral vectors substantially augmented the expression of molecules associated with osteogenesis (48). Additionally, the authors observed that the inhibition of IRE1α/XBP1 effectively counteracted the bortezomib-induced bone formation in mice, as evidenced by micro CT analysis. These findings provide compelling evidence for the pivotal role of the XBP1 signaling pathway in bone formation induced by PI (44).

### 4.3 XBP1 is highly expressed in PDLCs and is involved in alveolar bone formation

Periodontal ligament cells (PDLCs) are the cytological basis for periodontal tissue damage repair and regeneration (49), and their osteogenic potential is particularly critical. Disturbances in tissue homeostasis can initiate the osteogenic differentiation of PDLCs. RT-PCR of osteogenesis-induced PDLCs revealed that PDLCs undergo upregulation of ER stress levels during the osteogenic differentiation process and that XBP1 plays a role in it (50, 51).

BMP2 is a highly conserved functional protein of the TGF-β family, promoting osteogenesis (52, 53). It was shown by immunofluorescence that XBP1 highly expresses in human periodontal ligament cells (hPDLCs). At the same time, BMP2 could co-localize with XBP1 in hPDLCs and jointly improve the osteogenic ability of hPDLCs, suggesting that XBP1 has a positive role in the proliferation and osteogenesis of hPDLCs XBP1 have a positive role in both proliferation and osteogenesis of hPDLCs (54).

Lipopolysaccharide (LPS), a potent toll-like receptors (TLR) 4 agonist (55), has been shown to induce activation of the UPR in hPDLCs. When the UPR is activated, PERK, ATF6, and IRE1 are released from GRP78 to transduce the signal through the ER membrane to the cytoplasm and nucleus. Many studies have shown that LPS induces GRP78 expression in various cell types (56), activating the UPR. A previous study showed that qrt-PCR analysis of human periodontal ligament fibroblasts (hPDLFs) treated with LPS showed that LPS increased the expression of XBP1 mRNA level and promoted the splicing and activation of XBP1 in hPDLFs, thus participating in the regulation of the alveolar bone formation process (57).

It has also been shown in the literature that XBP1 is involved in a range of inflammation-related diseases by acting as a target gene for specific miRNAs. Exo-miR-205-5p inhibits inflammation in chronic periodontitis by targeting XBP1 (58). Whether it promotes osteoblast differentiation has not been reported.

## 5 Regulation of XBP1 in the immune microenvironment of bone remodeling

Bone and immune cells co-exist in the bone marrow cavity and share a common progenitor cell and a diversity of regulatory molecules. Immune cells regulate bone homeostasis, and bone cells, in turn, influence the proliferation and differentiation of immune cells (59). Tissue injury triggers an immediate immune response, and the initial immune response consists primarily of the innate immune system, including neutrophils, mast cells, monocytes, and macrophages. The later immune response consists primarily of the adaptive immune system, including T cells and B cells. Immune cells have two main effects on bone regeneration: the anabolic function of acute inflammation and the catabolic function of chronic inflammation (60). The following focuses on the regulatory role of XBP1 in several common immune cells.

Macrophages are innate immune cells in almost all tissues and play a key role in maintaining normal tissue homeostasis. During acute inflammation, macrophages promote the restoration of tissue homeostasis by phagocytosing invading microorganisms, amplifying the inflammatory response, and recruiting extra immune cells (61). When tissue damage is cleared, macrophages promote tissue regeneration by secreting anti-inflammatory factors, recruiting progenitor cells, and producing growth factors. This functional plasticity of macrophages is defined as macrophage polarization (62). Macrophages during inflammation are referred to as M1-type macrophages, whereas macrophages active in tissue regeneration are M2-type macrophages (29, 63). Numerous studies have demonstrated the critical role of macrophages in abnormal bone metabolism.

T cells belong to the adaptive immune system with great specificity and immune memory for their targets (64). T cells are divided into two categories according to their T cell receptors: αβ T cells and γδ T cells. αβ T cells are subdivided into CD4-positive (CD4+) and CD8-positive (CD8+) T cells. CD4+ T cells, also known as T helper (Th) cells, are divided into several subpopulations, including Th1, Th2, Th9, Th17, Th22, and follicular helper T cells (Tfh) and Tregs, which can be indirectly involved in clearing infections by regulating the activity of other immune cells (65). While CD8+ T cells are known as cytotoxic T lymphocytes (CTLs), CTLs target virus-infected cells to induce their death (64), both of which have dual functions of promoting and inhibiting regeneration. γδ T cells, a small subpopulation of T cells (66), are thought to have a regeneration-promoting role, but their mechanisms are unknown. Thus, the regulatory effect of T cells in bone regeneration may be associated with T cell subsets, cytokines, and other agents (60).

B cells belong to the adaptive immune system and are characterized by their ability to differentiate into plasma cells to produce antibodies. These antibodies permeate extracellular spaces, where they protect against infection (67). After their generation, B cells assume various functions in immune regulation and host defense (68). The development of B cells occurs in the bone marrow (BM) from hematopoietic stem cells (HSCs), progressing through a sequence of stages involving common lymphoid progenitors (CLP), progenitor (pro), precursor (pre), and immature B cells. Subsequently, their migration from the BM is triggered, and upon activation, B lymphocytes can either become terminal B cells that secrete Ig-secreting PCs or antigen-specific memory B cells that may eventually reenter the BM (69). There is a large body of literature that shows that B lymphocytes, through the secretion of inflammatory cytokines, matrix metalloproteinases, and RANKL, as well as the production of pathogenic autoantibodies, play a skeletal disease role (70).

### 5.1 Regulatory role of XBP1 in macrophages

#### 5.1.1 XBP1 promotes the production of pro-inflammatory cytokines

**Figure 3:**
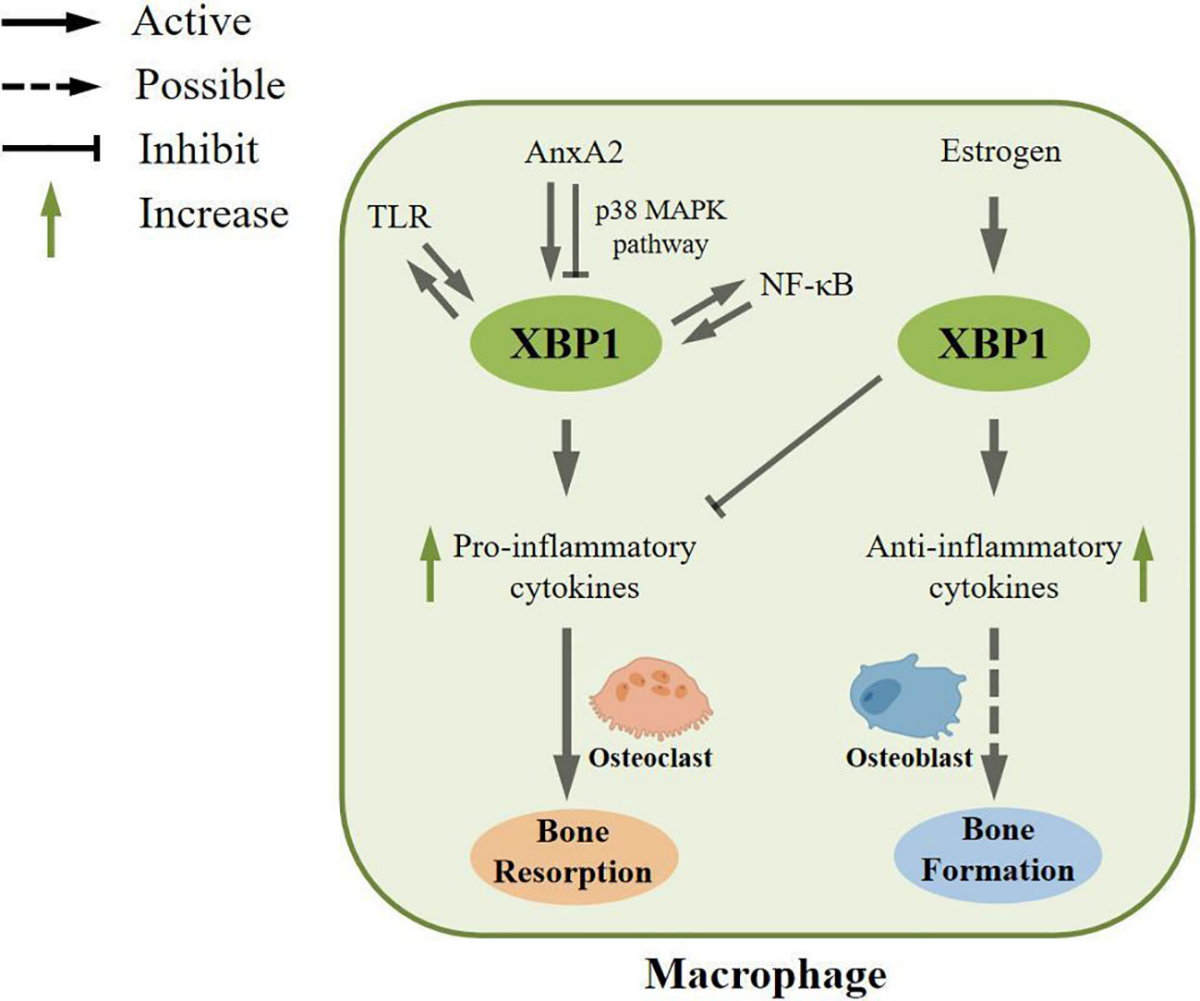
Regulatory role of XBP1 in macrophages. In macrophages, XBP1 can be activated by toll-like receptor (TLR) and maintain the TLR pathway. Second, XBP1 can be activated by Annexin A2 (AnxA2), which is involved in inflammatory responses, and AnxA2 can also inhibit XBP1 maturation through the p38 MAPK pathway. In addition, XBP1 can be activated by NF-κB pathway, and in turn, XBP1 can enhance the expression, phosphorylation, and nuclear transport of NF-κB pathway. These processes can increase the formation of pro-inflammatory cytokines, which could potentially be involved in bone resorption. In contrast, XBP1 can also be activated by estrogen to increase anti-inflammatory cytokines and inhibit the expression of pro-inflammatory cytokines, which may ultimately play an essential role in bone formation.

TLRs are a class of transmembrane proteins with a type I membrane structure, characterized by an ectodomain comprised of leucine-rich repeat sequences (LRRs) responsible for the recognition of pathogen-associated molecular patterns (PAMPs) (71, 72). In a scholarly study, the utilization of a Phos-tag reagent, which selectively binds to phosphorylated amino acid residues, was employed to investigate the activation of mouse J774 macrophages cultured in polyacrylamide gels containing Phos-tag acrylamide upon stimulation with the TLR agonist LPS. The presence of IRE1α was detected in these stimulated macrophages. Additionally, the activation of TLRs was also observed in primary macrophages derived from WT mice (C3H/HeOuJ) under LPS stimulation, as evidenced by the presence of TLR-activated XBP1 (20). XBP1 has been observed to fulfill a positive feedback function in macrophages, thereby preserving the integrity of the TLR pathway. Furthermore, the absence of *Xbp1* in mice resulted in impaired secretion of inflammatory mediators, as evidenced by ELISA and RT-PCR assays conducted on TLR4 or TLR2 agonist-stimulated macrophages. This impairment serves as a demonstration of XBP1’s role in augmenting the production of pro-inflammatory cytokines through TLR activation. In line with this discovery, the activation of XBP1 was also found to synergistically enhance the regulation of cytokines by TLR in M1-type macrophages (20) (**Figure 3**).

Annexin A2 (AnxA2) is a multicompartmental compartmentalized protein. It is thought to mediate the linker protein of the membrane-associated protein complex to regulate the recruitment of leukocytes and the release of inflammatory mediators (73, 74). Currently, a large body of literature shows that AnxA2 is associated with ER stress and is located upstream of XBP1 involved in the inflammatory response (75). Some scholars have used siRNA technology to silence AnxA2, and transcript and protein levels of XBP1 were significantly reduced in mouse alveolar macrophage cell line (MH-S) and THP-1 cells with silenced AnxA2 after *P.aeruginosa* infection, indicating that AnxA2 deletion can impair *P.aeruginosa*-induced macrophage ER stress. Original studies have shown that AnxA2 promotes the production of pro-inflammatory cytokines (76) and may play a role in M1-type macrophages. Moreover, overexpression of XBP1 in MH-S stimulated the transcription of TNFα, IL-1β, and IL-6 in response to *P.aeruginosa* infection, suggesting that XBP1 could stimulate the transcription of pro-inflammatory cytokines. However, by measuring the levels of p38 and phospho-p38, it was found that AnxA2 could inhibit the maturation of XBP1 through the p38 MAPK pathway, thereby regulating the formation of inflammatory cytokines (75).

The nuclear factor NF-κB pathway has long been considered the classic pro-inflammatory signaling pathway, mainly based on the role of NF-κB in the expression of pro-inflammatory genes, including cytokines, chemokines, and adhesion molecules (77). NF-κB dimers can bind to related target DNA sequences, called κB sites, to regulate gene expression (78). A large body of literature shows that NF-κB is associated with ER stress (79). XBP1 expression was downregulated in MH-S cells using siRNA technology. qPCR, immunoblot analysis, and imaging analysis showed that XBP1 enhanced NF-κB pathway’s expression, phosphorylation, and nuclear translocation. At the same time, this pathway can enhance the formation of pro-inflammatory cytokines (75).

#### 5.1.2 XBP1 promotes the production of anti-inflammatory cytokines

In contrast to the aforementioned findings, evidence suggests that the activation of IRE1α/XBP1 signaling by estrogen can impede M1-type macrophage polarization and potentially contribute to the anti-inflammatory mechanism. Estrogen, classified as a steroid hormone, assumes a crucial function in preserving immune homeostasis within the body (80). Predominantly present as estradiol (81), *in vivo* studies have demonstrated that estrogen can modulate the proliferation and differentiation of immune cells through its interaction with estrogen receptors located on these cells (82). Several researchers have administered estrogen stimulation exclusively to mouse macrophages and observed an upregulation in the expression of anti-inflammatory molecules, such as TGF-β and IL-10. Additionally, the activation of IRE1α signaling was significantly observed in macrophages following estrogen treatment, and this activation was hindered by the introduction of estrogen receptor antagonists. However, when both estrogen and IRE1α inhibitors were concurrently present, an upregulation in the expression of pro-inflammatory cytokines, including TNF-α and IL-6, was demonstrated. This experiment demonstrates that estrogen inhibits M1-type macrophage polarization by activating IRE1α/XBP1 signaling (29), thereby suppressing the inflammatory response.

### 5.2 Regulatory role of XBP1 in T cells

#### 5.2.1 XBP1 is upregulated in CD4+ T cells

Activated and differentiated helper T cells can secrete a large number of cytokines. It has been shown that after treatment with LPS and cecal ligation and puncture (CLP) *in vivo* and *in vitro* sepsis models, respectively, the ER stress levels and downstream markers increased in CD4+ T cells. At the same time, apoptosis of CD4+ T cells was significantly increased. This phenomenon was improved with the application of ER stress inhibitors (83). In addition, measuring the expression of IRE1α mRNA and protein in differentiated and reactivated Th2 cells *in vitro* using qPCR and Western blot showed that the IRE1α/XBP1 pathway is upregulated during helper T cell activation *in vitro*. Meanwhile, infecting C57BL/6 mice with the parasite Nippostrongylus brasiliensis demonstrated that the pathway is also active *in vivo* (84). Analysis of blood samples from patients with allergic rhinitis revealed that XBP1 might play an essential role in the development of Th2 polarization.

#### 5.2.2 XBP1 is upregulated in CD8+ T cells

**Figure 4:**
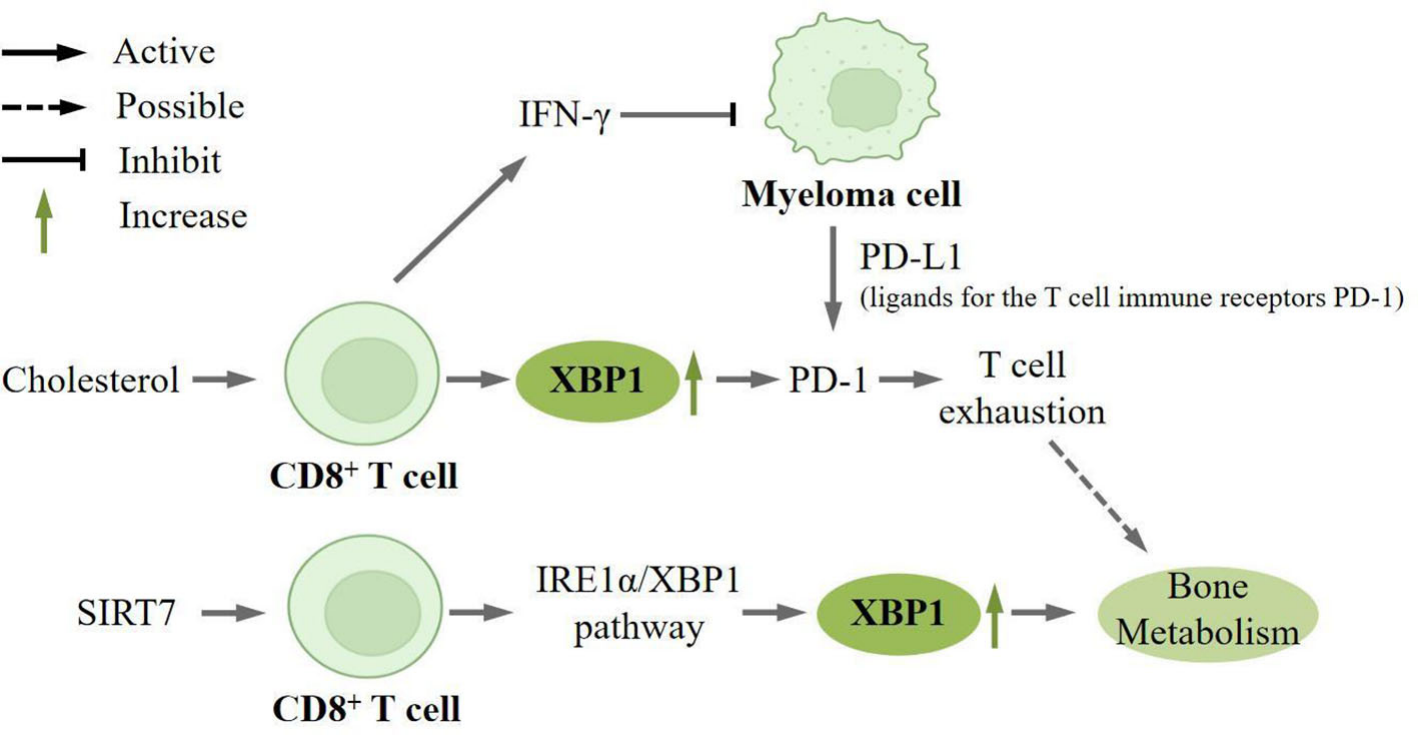
XBP1 is upregulated in CD8+ T cells. Cholesterol can induce high expression of XBP1 on CD8+ T cells, which in turn regulates the expression of the inhibitory receptor PD-1 on CD8+ T cells and induces T cell depletion.CD8+ T cells can produce IFN-γ, which directly inhibits the growth of myeloma cell. And myeloma cells can express PD-L1 (ligand for the T-cell immunoreceptor PD-1), which can directly lead to T-cell exhaustion, which may play a key role in bone metabolism. SIRT7 also promotes the expression of XBP1 in CD8+ T-cells, which is involved in bone metabolism.

Cholesterol is a key constituent of membrane lipids and plasma compartments that can play a role in the anti-tumor response of T cells (85). It has been recently shown that microarray analysis of CD8+ T cells under different cholesterol treatment conditions revealed that XBP1 was highly upregulated in cholesterol-treated CD8+ T cells. Meanwhile, *in vivo* and *in vitro* experiments revealed that knocking down XBP1 significantly downregulated Pdcd1 mRNA expression in CD8+ T cells. Moreover, in the group without knockdown of XBP1 expression, cholesterol treatment increased PD-1 expression, thus demonstrating that XBP1 is an important regulator of cholesterol-induced CD8+ T cell inhibitory receptor expression and T cell exhaustion. T cell exhaustion is characterized by impaired effector cell function, sustained expression of inhibitory receptors, and a distinct transcriptional profile compared to functional effector cells or memory T cells (86). Both extrinsic negative regulatory pathways, including immunomodulatory cytokines, and intrinsic negative regulatory pathways play a crucial role in the development of exhaustion. Through the ChIP assay, the researchers discovered that cholesterol can stimulate the expression of XBP1, which subsequently modulates the expression of inhibitory receptors on CD8+ T cells, such as PD-1, and induces T cell exhaustion (87). It has been reported that advanced T-cell failure is associated with loss of cytotoxicity in CD4+ and CD8+ T cell subsets that produce IFN-γ, and early *in vitro*, studies have shown that IFN-γ directly inhibits myeloma cell growth, and preclinical models support a positive role for CD8+ T-cell failure in myeloma progression (88) (**Figure 4**).

Sirtuins are a family of deacetylases that sense nutrients and regulate cellular homeostasis (89), of which SIRT7 is localized only in the nucleus and is associated with the maintenance of cellular homeostasis and adaptation to exogenous stress (90). Researchers found that knockdown SIRT7 significantly attenuated the expression of Tunicamycin (TM), an ER stress inducer, induced by these molecules. At the same time, overexpression of SIRT7 enhanced the upregulation of these molecules. Western blot and immunohistochemical staining analysis also demonstrated that SIRT7 could selectively activate the IRE1α/XBP1 branch (91). SIRT7 can promote the secretion of IRE1α-dependent tumor-promoting factors and alter the tumor immune microenvironment, thereby mediating tumorigenesis. It has been documented that SIRT7 is associated with the bone remodeling process (92).

### 5.3 Regulatory role of XBP1 in B cells

There are two distinct subpopulations of mature B cells, namely B-1 and B-2. B-1 B cells are predominantly located in the peritoneal cavity and are responsible for producing systemic natural IgM. Conversely, B-2 cells primarily participate in conventional T-cell-dependent IgM and IgG responses in peripheral lymphoid organs, but they also have the capability to migrate to the intestinal lamina propria and generate IgA. The activation of B cells into antibody-secreting PCs depends on the nature of the antigen, which can occur through either a T-cell-independent (TI) or a T-cell-dependent (TD) mechanism (93).

XBP1 is of significant importance in the differentiation of PCs and the secretion of Igs. Substantial research has consistently demonstrated the elevated expression of XBP1 in PCs, while the absence of XBP1 in mice results in the absence of PCs (93). Additionally, during the transition from B cells to PCs, there is a specific activation of the IRE1α and ATF6 branches of the UPR (94). These findings undeniably establish the essential role of XBP1 in PC differentiation. The differentiation process of B cells into PCs is governed by two transcription factors, namely B lymphocyteeinduced maturation protein 1 (BLIMP1) and XBP1. During the progression of PC differentiation, XBP1 acts as a regulatory factor downstream of Blimp-1 (95). Previous studies have demonstrated a significant decrease in mRNA and protein levels of XBP1, ATF4, and ATF6 in bone marrow PCs upon the inactivation of Prdm1, which encodes BLIMP1. Additionally, a ChIPseq assay conducted on *in vitro*-generated plasmablasts revealed that Blimp1 has the ability to activate ATF6, a known inducer of XBP1 (39). On the other hand, it can promote the formation of the active mature form of XBP1 by directly regulating *Ern1*, which encodes IRE1 (96).

In bone metabolism, B lymphocytes and PCs play a significant role by producing various cytokines and chemokines. These molecules have the ability to directly influence osteoblasts and also contribute to the regulation of bone remodeling and regeneration by modulating the bone microenvironment (69). Existing literature has provided evidence that both B cells and osteoblasts are capable of secreting OPG, which acts by inhibiting osteoclastogenesis through the blockade of the RANKL/RANK interaction. Among these cell types, PCs are particularly noteworthy as they are the most potent producers of OPG (30). The significance of OPG secretion by B cells in maintaining bone homeostasis was clearly demonstrated through studies involving mice with B-cell deficiency and osteoporosis (97). However, it has been reported that B cells and B cell-derived PCs in MM possess the ability to facilitate osteoclastogenesis, potentially attributed to the direct osteoclastogenic impact of RANKL expression (98–100) or the indirect promotion of bone resorption through IL-7 secretion (101, 102). Furthermore, B lymphocytes that have been pretreated with LPS hinder osteoclastogenesis in rat BMSCs by activating Notch signaling (69). It is evident that B lymphocytes exhibit diverse and sometimes conflicting influences on bone metabolism, contingent upon the specific physiological and pathological situation.

## 6 Discussion

In the context of bone metabolism, the UPR is initiated by the accumulation of misfolded proteins within the ER. Among the UPR pathways, the IRE1α/XBP1 is highly conserved. XBP1, a key component of this pathway, plays a critical regulatory role in bone metabolism. This article comprehensively elucidates the various roles of XBP1 in bone metabolism. Specifically, the involvement of XBP1 in osteoclast and osteoblast differentiation is now better understood. Notably, XBP1 has been shown to enhance osteoclastogenesis by binding to Nfatc1 in response to stimulation by RANKL/RANK. Additionally, XBP1 has the potential to indirectly enhance osteoclastogenesis by modulating the expression of RANKL on osteoblasts through positive regulation of PTH/PTHrP pathway. Moreover, XBP1 can facilitate osteoblast differentiation by promoting the expression of Osx. Furthermore, XBP1 can be activated by PI and actively participate in the process of bone formation. Notably, XBP1 may also exert its influence on periodontal tissues.

Interestingly, the regulatory effects of XBP1 on immune cells are multiple and often contradictory. In macrophages, XBP1 serves a dual function by facilitating the generation of pro-inflammatory cytokines and promoting macrophage polarization towards the M1 phenotype. Conversely, estrogen has the ability to activate XBP1, thereby inhibiting M1-type macrophage polarization. The impact of Th1 and Th2 cells on bone metabolism remains a subject of debate within the academic community. On one hand, Th1 cells have the capacity to activate osteoclasts, resulting in bone degradation. Conversely, both Th1 and Th2 cells produce a diverse array of cytokines that possess the ability to impede osteoclast differentiation, consequently promoting bone formation. Th17 cells have the ability to secrete active RANKL, thereby directly facilitating bone resorption. Considering the significant expression of XBP1 in T cells and its crucial involvement in bone metabolism, it is plausible to speculate that XBP1 may impact bone metabolism by influencing Th cells. Furthermore, XBP1 can modulate T cell dysfunction by regulating the expression of inhibitory receptors in CD8+ T cells and can be activated by SIRT7, potentially contributing to bone metabolism. In PCs, the expression of XBP1 is notably high. PCs have the capacity to significantly impact bone metabolism, both in terms of bone homeostasis and their potential to facilitate osteoclastogenesis. However, the precise molecular mechanism by which XBP1 regulates the aforementioned processes remains unclear.

In conclusion, further research is required to investigate: 1) the specific conditions that determine whether XBP1 activates osteoclast differentiation or osteoblast differentiation, and 2) the specific molecular mechanisms through which XBP1 regulates the differentiation of osteoblasts and osteoclasts by modulating immune cells. The current understanding of the relationship between XBP1 and bone metabolism is still limited. Exploring and comprehending the specific molecular mechanisms through which immune cells regulate bone metabolism via XBP1 could potentially complement and enhance therapeutic approaches and effectiveness in treating bone-related diseases. Consequently, investigating the regulatory function of XBP1 in bone metabolism may yield novel insights for clinical applications in this field.

## Author contributions

NM and LZ made significant contributions to the conception and design of this article. WL, ZZ, and JJ contributed to the drafting of the article. YZ, YF and TX revised the manuscript. All authors have read and agreed to the published version of the manuscript.
